# Screening and Identification of Transcription Factors Potentially Regulating *Foxl2* Expression in *Chlamys farreri* Ovary

**DOI:** 10.3390/biology11010113

**Published:** 2022-01-11

**Authors:** Shutong Fan, Xixi Li, Siyu Lin, Yunpeng Li, Huixin Ma, Zhifeng Zhang, Zhenkui Qin

**Affiliations:** 1 Ministry of Education Key Laboratory of Marine Genetics and Breeding, College of Marine Life Sciences, Ocean University of China, Qingdao 266003, China; fanshutong689@163.com (S.F.); lclixixi@163.com (X.L.); 15563886136@163.com (S.L.); 18738393963@163.com (Y.L.); mhx@stu.ouc.edu.cn (H.M.); 2 Laboratory of Tropical Marine Germplasm Resources and Breeding Engineering, Sanya Oceanographic Institution, Ocean University of China, Sanya 572000, China

**Keywords:** *Foxl2*, gonad, sexually dimorphic expression, transcriptional regulation, transcription factor, *Chlamys farreri*

## Abstract

**Simple Summary:**

*Foxl2* generally presents a sexually dimorphic expression pattern in animal gonads and is highly expressed in the ovary. However, few studies on the transcriptional regulation of *Foxl2* have been documented. To understand the transcriptional regulating of *Foxl2* high expression in the ovary, we used the Y1H system, a high throughput approach, for the first time to screen the transcription factors binding to the high transcriptional activity region of *Foxl2* promoter in Zhikong scallop (*Chlamys farreri*) gonads. In the present study, the highly transcriptional activity promoter sequence of *Cf*-*Foxl2* was determined at −1000~−616 bp and 11 candidate factors were verified to involve in *Cf-Foxl2* transcriptional regulation. Our findings provided valuable data for better understanding the specific transcriptional regulation mechanism of *Foxl2* in the ovary and would further assist in the breeding of aquacultural bivalves.

**Abstract:**

*Foxl2* is an evolutionarily conserved female sex gene, which is specifically expressed in the ovary and mainly involved in oogenesis and ovarian function maintenance. However, little is known about the mechanism that regulates *Foxl2* specific expression during the ovary development. In the present study, we constructed the gonadal yeast one-hybrid (Y1H) library of *Chlamys* *farreri* with ovaries and testes at different developmental stages using the Gateway technology. The library capacity was more than 1.36 × 10^7^ CFU, and the length of the inserted fragment was 0.75 Kb~2 Kb, which fully met the demand of yeast library screening. The highly transcriptional activity promoter sequence of *C. farreri Foxl2* (*Cf-Foxl2*) was determined at −1000~−616 bp by dual-luciferase reporter (DLR) assay and was used as bait to screen possible transcription factors from the Y1H library. Eleven candidate factors, including five unannotated factors, were selected based on Y1H as well as their expressional differences between ovaries and testes and were verified for the first time to be involved in the transcriptional regulation of *Cf-Foxl2* by RT-qPCR and DLR. Our findings provided valuable data for further studying the specific regulation mechanism of *Foxl2* in the ovary.

## 1. Introduction

FOXL2 (forkhead box L2) is a member of the forkhead superfamily which is characterized by the forkhead box/wing-helix DNA binding domain [1]. *Foxl2* was first studied in *Homo sapiens*, in which *Foxl2* mutation causes blepharophimosis-ptosis-epicanthus inversus syndrome (BPIS) and premature ovarian failure [2,3]. As an essential transcription factor, FOXL2 has been known to play roles not only in sex differentiation, ovarian cell differentiation, and function maintenance [4,5,6] but also in eyelid normal development [7], skeletal development [8], reactive oxygen species detoxification, and inflammatory processes [9].

Up to now, *Foxl2* has been revealed to be highly expressed in the ovaries of many animals, but weakly or even not expressed in the testis, showing a sexually dimorphic expression pattern [1,10,11,12]. In goat *Capra hircus*, loss of *Foxl2* leads to a sex reversal from female to male, in which the embryonic XX gonads develop into testes instead of ovaries [13]. In fish *Oreochromis niloticus*, *Foxl2* is revealed to involve in the production of estrogen maintaining ovarian differentiation [14], and its targeted deletion also causes female-to-male sex reversal [11]. In chicken *Gallus gallus*, *Foxl2* has been reported to play an important role in ovarian differentiation by antagonizing *Sox9* [15]. Up to now, more studies have focused on downstream genes regulated by FOXL2, and revealed several target genes related to steroidogenesis, proliferation, apoptosis, differentiation, and stress response, such as sex determination gene *Sox9* [16], *anti-Müllerian hormone* [17], estrogen receptor beta *Esr2* [18], and steroidogenesis-related gene *CYP19A1* [14,19,20], etc. However, studies on the transcriptional regulation of *Foxl2* have been rarely documented. Li et al. [21] found cadherin-associated protein β1 (CTNNB1) triggers *Foxl2* ectopic expression in the testis by directly binding the Tcf/Lef binding site of *Foxl2* promoter, and the CTNNB1-*Foxl2* pathway further induces the transformation of testicular Sertoli cells into ovarian granular cells in *Mus musculus*. Han et al. [22] reported that STAT3 may be involved in the transcriptional regulation of *Foxl2*, and the STAT3-*FoxL2* pathway plays an important role in HeLa cell apoptosis. A high motility group protein HMGA2 has been revealed to regulate metastases and epithelial-to-mesenchymal transition of chemoresistant gastric cancer by up-regulating *Foxl2* expression [23]. However, it is still largely unclear which TFs regulate *Foxl2*, and which TFs are involved in the *Foxl2* specifically expression in the ovary.

Bivalve is a kind of invertebrate with rich biodiversity and frequently occurred sexual reversal. In these species, *Foxl2* has also been revealed to present a sexually dimorphic expression pattern with significantly higher expression in ovary than that in testis, such as in Zhikong scallop *Chlamys farreri* [10], Yesso scallop *Mizuhopecten yessoensis* [24,25], freshwater mussel *hyriopsis cumingii* [26], and bay scallop *Argopecten irradians irradians* [27]. The Zhikong scallop *C. farreri*, one of the most important mariculture scallops in China, is a dioecious and sexually stable bivalve. In our previous research, we did not find the *M. musculus*-like CTNNB1 regulating *Foxl2* in *C. farreri*, suggesting that the transcriptional regulation of *Foxl2* in *C. farreri* may be different from that in *M. musculus*. To screen TFs regulating specific high expression of *Foxl2* in the ovary, we identified a promoter sequence of *Cf-Foxl2* with high transcriptional activity, constructed *C. farreri* gonadal yeast one-hybrid (Y1H) library using Gateway technology, and further verified TFs that may bind to this high transcriptional activity sequence through Y1H system. Outcomes of the present study would provide a theoretical basis to resolve the regulatory mechanisms of *Foxl2* in *C. farreri* and further assist in the breeding of aquacultural bivalves.

## 2. Materials and Methods

### 2.1. Animal Treatment and Sampling

Healthy male and female *C. farreri* with a mean shell height of 6.38 ± 0.33 cm were collected from the Nanshan Aquatic Products Market in Qingdao, China. Gonads (ovaries and testes) were dissected, rinsed with PBS (pH 7.4), immediately frozen in liquid nitrogen, and then stored at −80 °C for extractions of genomic DNA and total RNA.

According to the morphologic characteristics described by Liu et al. [28], the gonads were grouped into three stages based on histological structure and the gonadosomatic index (GSI = gonad weight/soft tissue body weight × 100): proliferative stage (GSI = 4.38 ± 0.22 for ovary and GSI = 4.77 ± 0.24 for testis), growing stage (GSI = 7.73 ± 0.33 for ovary and GSI = 8.87 ± 0.14 for testis), and mature stage (GSI = 9.65 ± 0.34 for ovary and GSI = 10.77 ± 0.94 for testis).

### 2.2. Cloning and Activity Assays of Foxl2 Promoter

The *Cf*-*Foxl2* gene sequence was obtained from the *C. farreri* genome (scaffold41309. 29:724895-741680) [29] and its transcription start site (TSS) was predicted using the online program FPROM. Available online: http://www.softberry.com/berry.phtml?topic=fprom&group=programs&subgroup=promoter (accessed on 10 January 2021). The genomic DNA of *C. farreri* ovary at the proliferative stage was prepared using Plant Genomic DNA Kit (Tiangen, Beijing, China). A 1905 bp promoter sequence (−1705~+200) of *Cf-Foxl2* was amplified using the ovarian cDNA as a template with the primers Fwd −1705/Rev +200 (Table S1).

**Figure 1:**
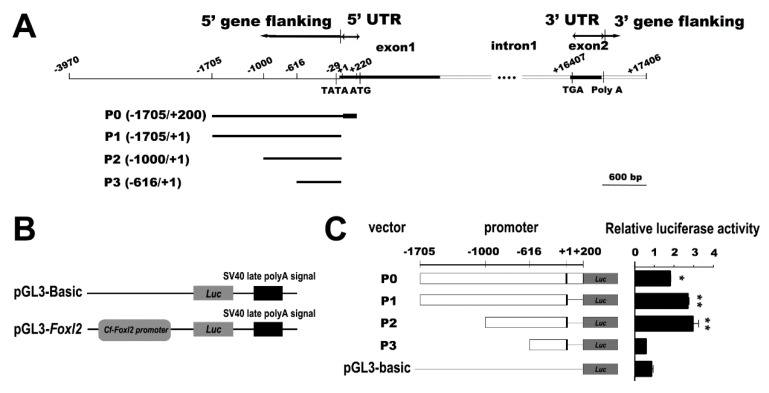
Identification of the high transcriptional activity promoter sequence of *Cf-Foxl2.* (**A**) Location diagram of *Cf-Foxl2* in genome; (**B**) schematic representation of the pGL3-*Foxl2* basic fusion constructs, and the pGL3-Basic construct; (**C**) activity of *Cf-Foxl2* promoter in HEK293T cells detected by DLR. pGL3-Basic: a cloning vector with only *Luciferase* (*Luc*), but without *Cf-Foxl2* promoter; ATG: initiation codon; TATA: TATA box; TGA: termination codon; TSS (+1): transcription start site. The luciferase activity of pGL3-Basic was set to “1.00” to calibrate the relative expression. * *p <* 0.05, ** *p <* 0.01.

A dual-luciferase reporter (DLR) assay system was employed to obtain the high transcriptional activity region in the promoter sequence. Briefly, the 1905 bp sequence was divided into four fragments (Figure 1A) and amplified with specific primers, respectively (Table S1). These PCR products were electrophoresis with 1.2% agarose gel, purified with SanPrep Column DNA Gel Extraction Kit (Sangon Biotech, Shanghai, China), cloned into the pMD19-T vector (TaKaRa, Beijing, China), and confirmed with Sanger sequencing. The sequenced cloning vector pMD19-T-*Foxl2* and the luciferase reporter vector pGL3-Basic (Promega, Madison, WI, USA) were linearized using QuickCut XhoI and HindIII restriction enzymes (TaKaRa, Beijing, China), and then these four fragments were respectively subcloned into pGL3-Basic and sequenced (Figure 1). HEK293T cells were cultured in a high-glucose Dulbecco’s Modified Eagle’s Medium (Hyclone, Logan, UT, USA) supplemented with 10% fetal bovine serum (Gibco, Grand Island, NE, USA) at 37 °C in a humidified environment with 5% CO_2_. The transient transfections of the constructed vectors (1000 ng in each assay) were performed in 24-well plates with HEK293T cell confluency of up to 90% and Invitrogen Lipofectamine^®^ 2000 Reagent (Thermo Fisher Scientific, Wilmington, NC, USA) diluted in Opti-MEM medium (Thermo Fisher Scientific, Wilmington, USA) according to the manufacture’s instruction as previously reported [30]. Luciferase activity was assayed at 48 h after transfection using the dual-luciferase reporter assay system (Promega, Madison, USA) according to the manufacture’s instruction. Relative luciferase activity = Firefly luciferase relative light units (RLU)/Renilla luciferase RLU. Three parallel transfections were conducted and the dual luciferase assay was repeated twice.

### 2.3. Construction of the C. farreri Gonadal Y1H cDNA Library

Total RNA was extracted from *C. farreri* male and female gonads of three stages using Trizol (Invitrogen, Carlsbad, CA, USA) according to the manufacturer’s protocol. The extracted total RNAs were analyzed with 1.2% agarose gel electrophoresis and Bioanalyzer 2100 (Agilent, Sant Clara, CA, USA), and quantified with NanoDrop^TM^ One/OneC (Thermo Fisher Scientific, Wilmington, USA). An equal mass of each sample was mixed together and the mRNA was separated and purified with the OLIGOTEX kit (Qiagen, Hilden, Germany) according to the manufacturer’s instructions. The double-strand cDNA was synthesized and ligated with attB1 adapters with the CloneMiner II cDNA Library Construction kit (Thermo Fisher Scientific, Wilmington, USA) according to the manufacturer’s instruction and ligated with attB1 adapters.

A cDNA library of *C. farreri* gonad was produced with the Gateway cloning technology [31]. Briefly, the normalized FL cDNA library was generated by BP Clonase^®^ II enzyme mix (Invitrogen, Carlsbad, USA) and cloned into pDONR222 vector and *Escherichia coli* DH10B (BP recombination reaction) for the construction of a primary library. Then, the primary library was further cloned into pGADT7-DEST vector and *Saccharomyces cerevisiae* Y187 with LR Clonase^®^ II enzyme mix (Invitrogen, Carlsbad, USA) to construct the secondary library (LR recombination reaction).

The quality of the Y1H library was validated through library capacity calculation (primary library), titer (secondary library), and inserted fragments inspection. The library capacity was calculated using the formula CFU/mL = Number of *E. coli* clones on the plate/50 μL × 1000 (dilution factor) × 10^3^ × total volume of library bacterial solution (mL). The titer was calculated with cells/mL = Number of *S. cerevisiae* clones on the plate x dilution factor. A total of 24 monoclonal colonies were randomly selected to test the size of inserted fragments and calculate the recombination rate of the library (percentage of clones with appropriate inserts to all clones). PCR amplification was performed using universal sequencing primers of the pGADT7-DEST vector (primers pGADT7-DEST-F/pGADT7-DEST-R, Table S1). The lengths of the inserted fragments were analyzed via 1.2% agarose gel electrophoresis.

### 2.4. Y1H Screening of the Potential Transcriptional Factors Regulating Cf-Foxl2 Expression

The Y1H assay was performed using the Matchmaker Gold Yeast One-Hybrid Library Screening System and Yeastmaker Yeast Transformation System (Clontech, Mountain View, CA, USA) described in the manufacturer’s instructions. The high transcriptional active region (−1000~−616 bp) of *Cf-Foxl2* promoter was amplified using the primer pairs Y1H-5/Y1H-3 (Table S1), and the amplified fragment was cloned into the pAbAi vector to generate a pAbAi-*Foxl2* bait vector. Then, the bait vector and p53-AbAi (positive control) were respectively linearized with the BstB I enzyme (NEB, Ipswich, USA) and integrated into the Y1HGold yeast strain, which was selected on synthetic defective SD/-Ura agar plate at 30 °C for 5 days. The recombinant Y1HGold strain was confirmed by PCR with the primer pairs Y1H-5/Y1H-3 (Table S1).

The competent Y187 yeast transformants containing the pAbAi-*Foxl2* bait vector were prepared as recipients, and the pGADT7-DEST prey plasmids (the secondary library) were transferred into them and screened on SD/−Leu agar plates with 100 ng/mL AbA at 30 °C for 5 days. Colonies with a >2 mm diameter were selected as positive candidates and amplified in SD/−Leu liquid medium; these vectors were then isolated with the Yeast Plasmid Extraction Kit (Tiangen, Beijing, China). Their sequences were identified by PCR with the primer pairs pGADT7-DEST-F/pGADT7-DEST-R (Table S1) and Sanger sequencing.

The TPM (transcripts per million) values of the candidate factors were retrieved from the *C. farreri* transcriptome database MolluscDB. Available online: http://mgbase.qnlm.ac/home (accessed on 9 December 2021) [32] and the differences between ovary and testis were calculated with DESeq2 [33]. The significant difference was set at the criteria:|Log_2_ FC| > 1 as well as *p* < 0.05. The candidate factors exhibiting sexual expression differences were selected for further verification.

In order to exclude false-positive results, the full-length CDS sequences of the candidate factors were amplified with gene-specific primers (Table S1), cloned respectively into pGADT7-DEST vector as preys, and then a second round Y1H were conducted as described above by transferring them into the same Y187 yeast recipients to confirm their interactions one-by-one. Meanwhile, the p53-AbAi vector was transformed into Y1HGold recipients as a positive control. All transformed Y1HGold yeast strains were cultured on SD/-Leu plates with 100 ng/mL AbA at 30 °C for 5 days, and colonies with a > 2 mm diameter were selected and analyzed. All transformation and screening were performed with three parallel repetitions.

### 2.5. Verification of Factors-Cf-Foxl2 Interaction with Dual-Luciferase Reporter (DLR) Assay

The full-length CDS sequences of candidate factors were amplified with primers (Table S1) and were cloned into pcDNA3.1 (+) to construct an expression vector, respectively. The DLR assay was conducted as described in 2.2 to determine the transactivation activity of the candidate factors on *Cf-Foxl2*. Two types of transfection systems were utilized: (i) single transfection: the recombinant pGL3-*Foxl2* vector containing the high transcriptional activity sequence detected in 2.2 was transfected into HEK293 cells alone; (ii) co-transfection: the pGL3-*Foxl2* vector was co-transfected with the constructed candidate factor-expression vector above (500 ng pGL3-*Foxl2*-Luc vector, 500 ng factor-expression vector) respectively. The pGL3-*Foxl2*-Luc + pcDNA3.1 (+) group was used to calibrate expression levels of different transfection systems.

### 2.6. RT-qPCR Analysis

The mRNA levels of the candidate factors in the gonads during the reproductive cycle were detected using RT-qPCR with specific primers (Table S1), and *Ef-1α* was used as a reference gene [24,34]. The reactions were carried out using SYBP Green Real-Time PCR Master Mix (Takara, Beijing, China) and the LightCycler^®^ 480 Real-time fluorescence quantitative PCR instrument (Roche, Basel, Switzerland). All reactions were performed in triplicate. The 2^–ΔΔCt^ method was used to analyze the mRNA expression level of these factors [35].

### 2.7. Statistical Analysis

All data were presented as the mean ± SEM from three samples and three technical replicates. Statistical analysis was tested using one-way ANOVA followed by Duncan’s test (SPSS software version 22.0, Chicago, IL, USA). For all analyses, statistical significance was set at *p* < 0.05, and extreme statistical significance was set at *p* < 0.001, respectively.

## 3. Results

### 3.1. Identification of Cf-Foxl2 Promoter with High Transcriptional Activity

The *Cf-Foxl2* gene sequence (scaffold41309. 29:724895-741680) was 21, 376 bp in full-length and consisted of 2 exons and 1 intron (Figure 1A). The conserved TATA box element in *Cf-Foxl2* promoter was predicted at 29~−22 nt upstream of the TSS (Figure 1A), the GC frame at −43~−38 nt, and −72~−63 nt, as well as the octamer ATATACAAAC at −26~−17 nt (Figure S1).

A 1905 bp promoter sequence (−1705/+200 bp) of *Cf-Foxl2* was amplified using the primers fwd −1705 and rev +200 (Table S1), and then a series of double-luciferase reporter vectors containing the different fragments of *Cf-Foxl2* promotor (Figure 1B) were constructed and transiently transfected into HEK293T cells to determine the high transcriptional activity region. The results showed that the transcriptional activities of the promoter fragments, P1 (−1705/+1) and P2 (−1000/+1) were significantly higher than that of P0 (−1705/+200), but no significant difference existed between P1 and P2 (Figure 1C). In addition, the promoter activity of P3 (−616/+1) was significantly decreased compared to the other three fragments (*p* < 0.05). Therefore, the region (−1000~−616 bp) in the *Cf-Foxl2* promoter sequence was determined as a high transcriptional activity region which would be used as a target sequence for screening the related transcription factors in the Y1H system.

### 3.2. Screening and Verification of Candidate Factors Interacting with Cf-Foxl2 Promoter Using Y1H

The extracted total RNAs from each of the ovaries and testes of *C. farreri* at the different stages were mixed equally as one sample, and its RIN value was determined to be 7.9 (Figure S2A). The visual assessment of the electropherogram revealed that no sign of degradation occurred (Figure S3). High-quality mRNA was purified with the greatest intensity between 0.75 and 2 kb (Figure S2B), and the synthesized double-stranded cDNA using the Gateway technology was uniformly dispersed without disproportionate enrichment of specific fragments (Figure S2C), suggesting a broad size range of the double-stranded cDNA and suitable parameters for library construction.

A three-frame cDNA library of *C. farreri* gonads (primary library) was constructed by connecting cDNA to the pGADT7-DEST vector using homologous recombination. The recombinant vectors were electroporated into *E. coli* DH10B, the inserted fragments were determined with a size range from 0.75 Kb bp to 2 Kb in length, a recombination efficiency was obtained 100% (Figure S4A) and the library capacity was 1.2 × 10^7^ CFU/mL (Figure S4C). The inserted fragments of the *S. cerevisiae* Y187 yeast library (secondary library) ranged from 0.75 Kb bp to 2 Kb in length, with a recombination efficiency of 100% (Figure S4B) and a library capacity of 1.36 × 10^7^ CFU/mL (Figure S4D). The results suggested that the gonadal Y1H library was successfully constructed.

Y1H screenings were conducted using the *Cf-Foxl2* high transcriptional activity sequence (−1000~−616) as bait. A total of 348 positive monoclonal colonies were obtained, which was 145 for the first screening and 203 for the second one. Sixty-four coding sequences were identified from the screening, of which 10 sequences were shared in both screenings. Furthermore, eight functionally unannotated sequences were obtained (Table S2). The 64 genes corresponding to 64 positive insertion sequences were the candidate factors interacting with *Cf-Foxl2.* Further, based on *C. farreri* gonadal transcriptome [36], 25 out of the 64 candidates were differentially expressed (|Log_2_FC| ≥ 1, *p* < 0.05) between testes and ovaries (Table S3). Among these different expression genes, expression levels of 15 genes were significantly higher in ovaries while 10 of them were significantly higher in testis. Finally, in view of their functional annotation seven gonadal differentially expressed genes (five highly expressed in ovary, two highly expressed in testis) as well as five unannotated and potentially novel genes screened by Y1H were selected for the Y1H reverse verification (Table 1).

**Figure 2:**
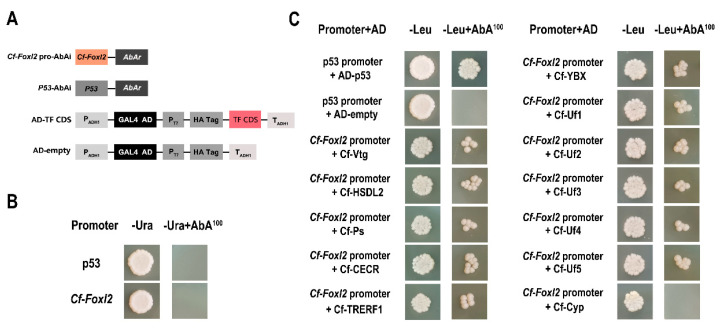
The interaction between selected factors and the *Cf-Foxl2* promoter assayed by Y1H reverse verification. (**A**) Schematic representation of vectors used in Y1H assays. (**B**) Auto-activation inspection of *Cf-Foxl2* promoter on SD/−Ura medium with 100 ng/mL Aba. (**C**) Physical interactions between selected factors and the *Cf-Foxl2* promoter using Y1H analysis on SD/−Leu medium with 100 ng/mL Aba.

To further confirm the interaction between the candidate factors and *Cf-Foxl2* promoter, we ligated the full-length cDNA sequence of the 12 selected genes respectively into the pGADT7 yeast plasmid and transformed it to the Y1HGold [*Foxl2*-AbAi] receptor cells again. The Y1H results showed that Y1H Gold yeast cells containing pGADT7-Vtg, HSDL2, Ps, CECR, YBX, TRERF1, Uf1, Uf2, Uf3, Uf4, and Uf5 could grow on SD/-Leu/AbA^100^ selective medium, which means these 11 selected factors can interact with the *Cf-Foxl2* promoter in the Y1H system (Table 1, Figure 2). However, the pGADT7-Cytochrome P450 1A1 could not (Table 1, Figure 2).

### 3.3. Identification of the Candidate Factors Regulating Cf-Foxl2 Transcription Using Transient Transfection

**Figure 3:**
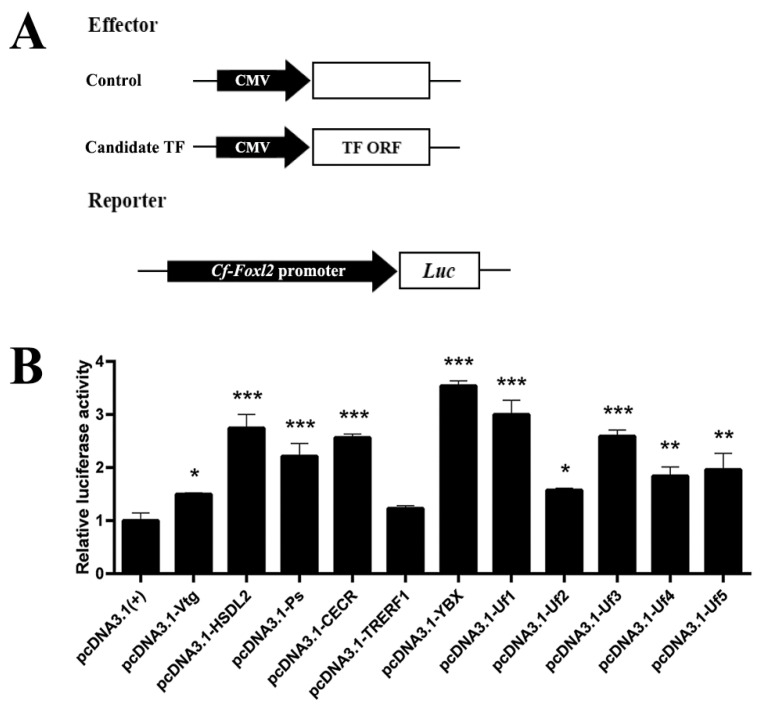
*Cf-Foxl2* transcriptional regulation by the candidate factors detected with transient transfection. (**A**) Schematic representation of the report vector and effector vector compositions. (**B**) The *Cf-Foxl2* transcription regulation in the HEK293T cells transfected transiently with the report and effect vectors. 1: PGL3-*Foxl2*-*Luc* + pcDNA3.1 (+); 2: PGL3-*Foxl2*-*Luc* + pcDNA3.1-*Vtg*; 3: PGL3-*Foxl2*-*Luc* + pcDNA3.1-*HSDL2*; 4: PGL3-*Foxl2*-*Luc* + pcDNA3.1-*Ps*; 5: PGL3-*Foxl2*-*Luc* + pcDNA3.1-*CECR*; 6: PGL3-*Foxl2*-*Luc* + pcDNA3.1-*TRERF1*; 7: PGL3-*Foxl2*-*Luc* + pcDNA3.1-*YBX*; 8: PGL3-*Foxl2*-*Luc* + pcDNA3.1-*Uf1*; 9: PGL3-*Foxl2*-*Luc* + pcDNA3.1-*Uf2*; 10: PGL3-*Foxl2*-*Luc* + pcDNA3.1-*Uf3*; 11: PGL3-*Foxl2*-*Luc* + pcDNA3.1-*Uf4*; 12: PGL3-*Foxl2*-*Luc* + pcDNA3.1-*Uf5*; *Luc*: *luciferase*. The luciferase activity of pGL3-*Foxl2*-Luc + pcDNA3.1 (+) was set to “1.00” to calibrate the relative expression. All data are presented as the mean ± SD (*n* = 3). * *p <* 0.05, ** *p <* 0.01, *** *p* < 0.001.

The *Foxl2* transcription regulated by the 11 candidate factors was further detected in HEK293T cells by transient co-transfection with the reporting vector (pGL3-Basic) containing the *Cf-Foxl2* high transcriptional activity region as well as the effector vector pcDNA3.1 (+) containing full-length ORF of the factors. The results (Figure 3) showed that all these TFs could activate the *Foxl2* transcription, and the activation intensity was significantly higher than that of control cells with only the recombinant plasmid PGL3-*Foxl2*-*Luc* and without transcription factors. Among the transcription factors, Y-box presented the highest activation, which was 3.5-fold higher than that of the control. Five unannotated factors also significantly increased the *CF-Foxl2* expression, although their transcriptional activations were different. Interestingly, Vitellogenin-4 was identified as a transcription factor in the high transcriptional activity region of *Cf-Foxl2*, which significantly increased the *Foxl2* transcription by about 1.5-fold than that of the control.

### 3.4. Expression of the Candidate Factors in the Gonads of C. farreri during the Reproductive Cycle

**Figure 4:**
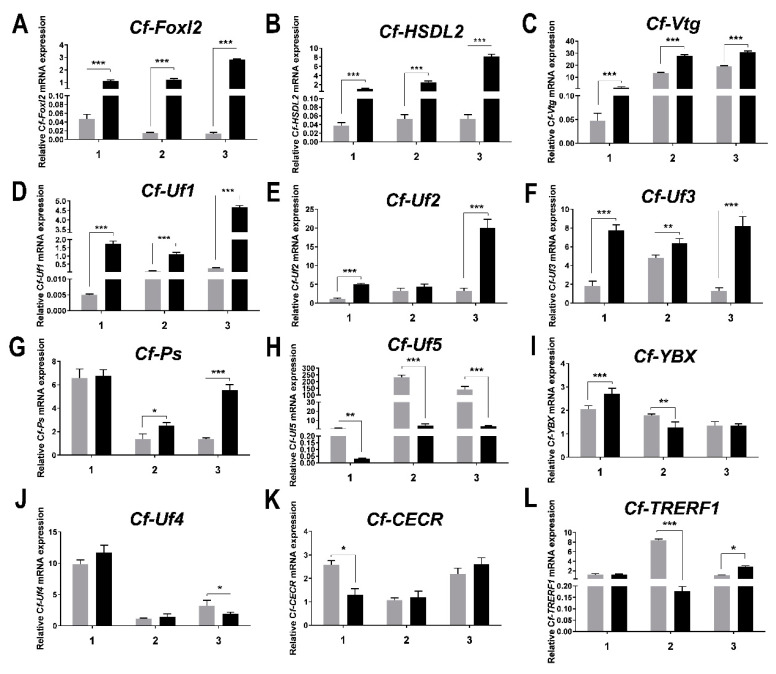
The mRNA expression levels of the candidate factors and *Cf-Foxl2* in the gonads of *C. farreri* at different stages. Gray bars and black bars: the expression levels in testis and ovary, respectively. 1: proliferative stage, 2: growing stage, 3: mature stage. (**A**–**G**), (**I**,**J**): ovarian with the lowest expression were set to “1.00” to calibrate the relative expression; (**H**,**K**,**L**): testis with the lowest expression were set to “1.00”. Values are presented as the mean ± SD (*n* = 3). * *p* < 0.05, ** *p <* 0.01, *** *p* < 0.001.

The RT-qPCR results (Figure 4) showed that these 11 candidate factors and *Cf-Foxl2* were differentially expressed between testes and ovaries (*p* < 0.05). Among them, six target genes (*Cf-HSDL2*, *Cf-Vtg*, *Cf-Uf1, Cf-Uf2, Cf-Uf3*, and *Cf-Ps*) were expressed significantly higher in the ovary than in the testis (Figure 4B–G). *Cf-HSDL2* mRNA levels exhibited the highest significant differences between gonads during the annual reproductive cycle and the mRNA level in the ovary at the mature stage was 151.9-fold higher than that in the testis at the same stage (Figure 4B). The *Cf-Uf1* expression (Figure 4C) showed a similar trend with that of *Cf-Foxl2* (Figure 4A), and the *Cf-Uf1* mRNA level was 18.79-fold higher in the ovary at maturity stage compared to the contemporaneous testis (Figure 4D). On the contrary, *Cf-Uf5* expression levels presented significantly higher expressed in *C. farreri* testis than that in the ovary (Figure 4H), with the highest expression level of 56.8-fold higher in the mature testis than that in the ovary at the same stage. In addition, the expression levels of four genes (*Cf-YBX*, *Cf-Uf4*, *Cf-CECR*, and *Cf-TRERF-1*) showed inconsistent differences between sex at different stages (Figure 4I–L).

## 4. Discussion

*Foxl2* generally presents a sexually dimorphic expression pattern in animal gonads and is highly expressed in ovaries [1,10,11,12]. To understand the transcriptional regulation of *Foxl2* high expression in animal ovaries, we used the Y1H system, a high-throughput approach, for the first time to screen the transcription factors binding to the high transcriptional activity region of *Cf-Foxl2* promoter in *C. farreri* gonads.

### 4.1. A High Transcriptional Activity Region of Cf-Foxl2 Promoter Is Identified for Conducting the Y1H Assay

Based on the prediction by FPROM software, we found that *Foxl2* promoter of *C. farreri* possessed several core promoter elements commonly existed in the structural gene of eukaryotic organisms [37], such as the TATA box at −29~−22 nt, the GC frame at −43~−38 nt and −72~−63 nt, as well as the octamer ATATACAAAC at −26~−17 nt. Usually, tissue-specific expression regulatory elements of a structural gene are the sequences outside the core promoter elements. Therefore, researchers have mostly selected the 2 kb region upstream of ATG for screening the key transcription factors regulating the target gene transcription [38,39,40]. In the present study, we cloned a 1.9 kb sequence upstream of ATG from the *Cf-Foxl2* gene (Figure 1A). Considering the technical requirements of Y1H that the optimum length of bait sequence used in Y1H was about 500 bp [41,42], we generated four fragments of the *Cf-Foxl2* promoter used for the transcriptional activity detection with a DLR system, P0 (−1705/+200) with a partial sequence of the first exon, P1 (−1705/+1) without the sequence of the first exon, P3 (−616/+1) with only the core promoter sequence. Considering that the truncation of *Cf-Foxl2* promoter should avoid the separation of transcription factor binding sites predicted by PROMO. Available online: http://alggen.lsi.upc.es/cgi-bin/promo_v3/promo/promoinit.cgi?dirDB=TF_8.3/ (accessed on 16 June 2021), JASPAR. Available online: https://jaspar.genereg.net/ (accessed on 16 June 2021) and GPMiner. Available online: http://gpminer.mbc.nctu.edu.tw/ (accessed on 16 June 2021) software, P2 (−1000/+1) was truncated between P1 and P3. The results (Figure 1C) showed that LUC activity of P2 (−1000/+1) was the highest and had no significant difference with P1 (−1705/+1), while significantly higher than that of P0 (−1705/+200) and P3 (−616/+1). Because the sequence length of P2 (1000 bp) was still not optimal for Y1H screening and the subsequent analysis, we further compared the transcriptional activities of P2 and P3 and proposed that the highly transcriptional region of *Cf-Foxl2* promoter should be located at −1000~−616 nt (Figure 1C).

### 4.2. High Quality C. farreri Gonadal Y1H Library Is Constructed

The yeast one-hybrid system (Y1H) provides a convenient gene-centered (DNA to protein) approach to identify transcription factors that can bind the DNA sequence of interest genes [43]. A high-quality cDNA library is determined by the crucial elements including the length of inserted fragments, the recombination and transformation efficiency, and the library capacity [44]. In the present study, the total RNA mixture from *C. farreri* gonads at different developmental stages obtained an RIN value of 7.9 (Figure S2A), and the visual assessment of its electropherogram exhibited no sign of the reduction in signal magnitudes (Figure S3), indicating the intactness of RNA. ds-cDNA gel electrophoresis examination showed the greatest cDNA intensity between 0.75 Kb and 2 Kb (Figure S2B), suggesting a broad range of sizes and was suitable for Y1H library generation. The primary library storage capacity was over 1.2 × 10^7^ CFU/mL (Figure S4C), and the secondary library storage capacity was more than 1.36 × 10^7^ CFU/mL (Figure S4D), which surpassed the minimum requirement of 1 × 10^6^ CFU for an informative yeast cDNA library [45]. Moreover, in randomly selected clones, the length of the inserted fragments was ranged from 0.75 Kb to 2 Kb and was coincident with the library. Our data indicated that the quality of the constructed gonadal Y1H library of *C. farreri* was high and fully met the demand of yeast library screening.

### 4.3. Specific Transcription Factors Potential Involved in the Regulation of Foxl2 Expression in C. farreri Gonad

Tissue-specific transcription factors play important roles in regulating the expression and biological functions of target genes. In the present study, we verified for the first time that eleven candidate factors interacted with *Cf-Foxl2* high transcription activity sequence (−1000~−616 bp) by Y1H (Figure 2) reverse verification, and significantly up-regulated the *Cf-Foxl2* expression in HEK293T cells by the transient transfection assay (Figure 3). Based on the transcriptome data at the mature stage (Table 1) and the RT-qPCR results (Figure 4) at different developmental stages of *C. farreri* bisexual gonads, expressions of these 11 candidate genes were all presented significantly different between ovaries and testes. Among them, six genes, including *Hydroxysteroid dehydrogenase-like protein 2* (*HSDL2*), *Vitellogenin-4* (*Vtg*), *Protein singed* (*Ps*) and three unannotated factors (*Uf1*, *Uf2*, and *Uf3*) exhibited higher expression in the ovary than that in the testis during the annual development of *C. farreri*. As a new member of the steroid dehydrogenase family, HSDL2 is considered to be involved in lipid metabolism, and the occurrence, proliferation, development of cancer cells [46,47]. In the present study, we revealed that *HSDL2* mRNA exhibited the highest level in the ovary, which was 154.9-fold higher than that in testis at the mature stage (Figure 4B), and its expression trend in the bisexual gonads during the annual development in *C. farreri* was similar to that of *Cf-Foxl2* (Figure 4A). Meanwhile, HSDL2 also presented the higher transcription activation of *Cf-Fxol2*, which was 2.7-fold higher than that of the control (Figure 3), suggesting HSDL2 is an important transcription factor to specifically regulate *Foxl2* higher expression in the *C. farreri* ovary. Intriguingly, three unannotated factors, Uf1, Uf2, and Uf3, were firstly verified to interact with *Cf-Foxl2* high transcriptional activity sequence and significantly increased the *Cf-Foxl2* expression level (Figure 3 and Figure 4). In particular, the transcription activation of *Cf-Uf1* on *Cf-Foxl2* was 3.63-fold higher than that of the control (Figure 3), and the *Cf-Uf1* expression pattern in the bisexual gonads during annual development (Figure 4D) was also consistent with that of *Cf-Foxl2*, suggesting that *Cf-Uf1* might also play an important role in *Cf-Foxl2* transcription regulation. Moreover, in this study, VTG (Vitellogenin-4), commonly considered as a nutritional source as well as participating innate immune defense [48,49,50], was first discovered to possibly act as a transcription factor of regulating *Cf-Foxl2* expression. However, further research is needed to illustrate detailed mechanisms.

This study also identified an unannotated factor (Uf5) which was significantly higher expressed in *C. farreri* testis than that in the ovary during the annual development (Figure 4H). Considering that *Cf-Uf5* was the only special case among the 11 transcription factors and its activation ability was also not the most prominent (Figure 3), it is speculated that *Cf-Uf5* may not play a decisive role in *Cf-Foxl2* expression.

The situation of the other four factors (YBX, Uf4, CECR, and TRERF-1) was more complicated, in which the expression levels of these genes presented inconsistent sex differences in bisexual gonads at different developmental stages (Figure 4I–L). More rigorous experiments are needed to analyze their regulatory roles later.

Previously, several transcription factors regulating *Foxl2* specific expression have been revealed, such as HMGA2 in chemotherapy-resistant gastric cancer of *M. musculus* [23]; STAT3 in apoptosis of *M. musculus* HeLa cells [22,39]; while the regulation of *Foxl2* transcription in gonads has only been reported in *M. musculus*, in which CTNNB1 was found to induce the transformation of Sertoli cells into granular cells by activating ectopic expression of *Foxl2* in the testis of *M. musculus* [21]. In this study, we identified 64 gene-encoding proteins potentially interacting with the *Cf-Foxl2* high transcription activity sequence by YIH library screening. Unfortunately, we did not find the transcription factors as reported. In addition, we cloned the *CTNNB1* sequence of *C. farreri*, and carried out transient co-transfection of CTNNB1 and *Cf-Foxl2* promoter sequence to test whether CTNNB1 could regulate *Cf-Foxl2* transcription. The results showed that CTNNB1 did not change the *Cf-Foxl2* expression level (Figure S5) and thus did not play a regulatory role in *C. farreri*. Furthermore, we used predictive software (PROMO, JASPAR and GPMiner) to try to find these reported transcription factors [21,22,23,39] in the 1.9 kb promoter of *Cf-Foxl2*, but only the STAT3 transcription factor was identified. Since the STAT3 binding site was predicted to be outside the *Cf-Foxl2* high activity region obtained in this study, and its expression level was not different between ovary and testis based on the gonadal transcriptome data of *C. farreri*, further experimental verification was not performed. In conclusion, the specific transcription factors regulating *Foxl2* expression should be species specific and tissue specific.

## 5. Conclusions

In the present study, a high transcriptional activity region of the *Cf-Foxl2* promoter was identified to be at −1000 to −616 bp. A number of transcription factors potentially involved in the specific regulation of *Foxl2* expression in the *C. farreri* gonad were screened using a high-quality *C. farreri* gonadal Y1H library constructed with Gateway technology. Eleven candidate factors were verified to significantly up-regulate *Cf-Foxl2* transcription levels, and HSDL2 and Uf1 may be important for the high expression of *Cf-Foxl2* in the ovary. Our data are meaningful to better understand the specific transcriptional regulation mechanism of *Foxl2* in the ovary.

## Figures and Tables

**Table 1:** Function of the candidate factors and their expression difference between ovaries and testes.

No	Candidate Factors	Function	Log_2_FC
1	Vitellogenin-4 (Vtg)	Lipid transport and storage; antioxidant activity.	−9.4367
2	Cytochrome P450 1A1 (Cyp)	Participates in the metabolism of various endogenous substrates, including fatty acids, steroid hormones, and vitamins.	−7.4725
3	Hydroxysteroid dehydrogenase-like protein 2 (HSDL2)	Participates in the physiological process of female sex differentiation and the generation and maintenance of secondary sexual characteristics.	−7.3104
4	Protein singed (Ps)	Acts as an actin binding protein; It is involved in setae and hair generation, cell differentiation and oogenesis.	−3.2786
5	Cat eye syndrome critical region protein 5 (CECR)	Participates in ocular development through the formation of ISWI chromatin complexes.	−3.0421
6	Unannotated factor 1 (Uf1)	-	#
7	Unannotated factor 2 (Uf2)	-	#
8	Unannotated factor 3 (Uf3)	-	#
9	Unannotated factor 4 (Uf4)	-	#
10	Unannotated factor 5 (Uf5)	-	8.2517
11	Transcriptional-regulating factor 1 (TRERF1)	Activation of *CYP11A1* transcription; it binds to the progesterone receptor.	1.1161
12	Y-box factor homolog (YBX)	Male gonadal development; spermatogenesis.	0

“-”: function unknown; LogFC: gene expression differences of *C. farreri* gonads in transcriptome database calculated using the formula Log_2_ (^testis expression TPM^/_ovarian expression TPM_); negative value: highly expressed in ovary; positive value: highly expressed in testis; 0: no difference between ovary and testis; #: no information from the transcriptome database.

## Data Availability

The data presented in this study are available in this article or Supplementary Materials.

## Supplementary Materials

The following are available online at https://www.mdpi.com/article/10.3390/biology11010113/s1, Figure S1: Schematic diagram of upstream elements of Foxl2 core promoter region of Zhikong scallop *C. farreri*; Figure S2: The synthesis, purification, and double-stranded cDNA; Figure S3: Electropherogram of *C. farreri* total RNA sample; Figure S4: The *C. farreri* gonadal Y1H library construction and quality test; Figure S5: The detection of CTNNB1 regulating *Cf-Foxl2* transcription by transient transfection; Table S1: Summary of primers used in this study; Table S2: Annotation of the positive insert sequences in yeast one-hybrid screening; Table S3: Function and sex difference information of the candidate transcription factors; Table S4: Relative bioluminescence intensity of *Cf-Foxl2* promoter transcriptional activity detected with DLR; Table S5: Relative bioluminescence intensity of factors-*Cf-Foxl2* interaction verification detected with DLR.
