# Supplementary material for: Screening and Identification of Transcription Factors Potentially Regulating Foxl2 Expression in Chlamys farreri Ovary
Source: Biology (Basel). 2022 Jan 11;11(1):113. doi: 10.3390/biology11010113 (PMC8772818; doi:10.3390/biology11010113)
Supplement: Supplementary file 1 [file biology-11-00113-s001.zip › biology-1525550-supplementary.pdf]

Screening and identification of transcription factors regulating *Foxl2* high expression in *Chlamys farreri* ovary

Shutong Fan [1], Xixi Li [1], Siyu Lin [1], Yunpeng Li [1,2], Huixin Ma [1], Zhifeng Zhang [1,2,*] and Zhenkui Qin [1*]

[1] Ministry of Education Key Laboratory of Marine Genetics and Breeding, College of Marine Life Sciences, Ocean University of China, Qingdao 266003, China
[2] Laboratory of Tropical Marine Germplasm Resources and Breesing Engineering, Sanya Oceanographic Institution, Ocean University of China, Sanya 572000, China
* Corresponding author: qinzk@ouc.edu.cn; zzfp107@ouc.edu.cn

Table S1. Summary of primers used in this study

Name	Primer sequence (5'-3')	fragment position（bp）	amplicon size（bp）
	For *Foxl2* promoter-LUC vector construction		
Fwd-1705	CTCGAGGGCTTCATACACTAGAGGCG	-1705 ~ +200	1905
Rev+200	AAGCTTATTATCACCAAATCCCTCGG		
Fwd-1705	CTCGAGACTCCTCCGTTGATGTTGAC	-1705 ~ +1	1706
Rev+1	AAGCTTATTATCACCAAATCCCTCGG		
Fwd-1000	CTCGAGAGAAACACAGAATCTTATCC	-1000 ~ +1	1001
Rev+1	AAGCTTATTATCACCAAATCCCTCGG		
Fwd-616	CTCGAGATACTCTGGTCCAAGTCCTG	-616 ~ +1	617
Rev+1	AAGCTTATTATCACCAAATCCCTCGG		
	Universal sequencing primers for pGADT7-DEST vector		
pGADT7-DEST-F	TAATACGACTCACTATAGGGCGAGCGCCGCCATG		
pGADT7-DEST-R	GTGAACTTGCGGGGTTTTTCAGTATCTACGATT		
	For amplification of *Foxl2* bait sequence		
Y1H-5	AAGCTTCCCTTGGGTCTGTTGTATTC	-1000~-616	385
Y1H-3	CTCGAGGCGGATGTATGATGGGACGA		
	For amplification of candidate factors CDS		
Vtg ORF-F	CGCCATATGATGTTCCGATTTCTGATGCTG		

Vtg ORF-R	CCGCTCGAGGCCCATACAAAGCCAAGC
Cyp ORF-F	CGCCATATGATGGGTGTACTAGAGGGGAT
Cyp ORF-R	CCGCTCGAGCATGGTGGGCCTCGCAATCA
HSDL2 ORF-F	CGCGGATCCTGTCCACACAATCCAGTAAT
HSDL2 ORF-R	CCGCTCGAGTTATCTCTTGACCCGGAATC
Protein singed ORF-F	CGCGGATCCATGTCGCAGTCTAATGGATG
Protein singed ORF-R	CCGCTCGAGTTAGAACTCCCAGTATGTAT
CECR ORF-F	CGCGGATCCTGGAGAGGAGGAATACTTTT
CECR ORF-R	CCGCTCGAGCATGTCGCCACGCGGAAGTT
YBX ORF-F	CCGGAATTCATGTCCGATTCCGAAAAACA
YBX ORF-R	CGAGCTCTTACTCGGCCTGCTCGCCTC
TRERF1 ORF-F	CCGGAATTCACTGACTCTACCACAGGATT
TRERF1 ORF-R	CGAGCTCCATCTGTTGGATAATGTCTC
Cf-Uf1 ORF-F	CGCGGATCCATGTTGCGGAGACTGGCG
Cf-Uf1 ORF-R	CCGCCGCTCGAGTTTCCCAGTAATATAAGC
Cf-Uf2 ORF-F	CGCGGATCCAGGTACCTACTCATGGAGATCA
Cf-Uf2 ORF-R	CCGCTCGAGACTAAGAAGTTCCAGGGCTGTA
Cf-Uf3 ORF-F	CGCGGATCCGGGCCCCAACGACATTCT
Cf-Uf3 ORF-R	CCGCCGCTCGAGACGGTGATTCTGCATAGT
Cf-Uf4 ORF-F	CGCGGATCCATGGTTCGTGTATTTGTTCAGA
Cf-Uf4 ORF-R	CCGCTCGAGTCACGAAGCACACGTGACCT
Cf-Uf5 ORF-F	CGCGGATCCATGCATCGAGCGTACCAGCC
Cf-Uf5 ORF-R	CCGCTCGAGTCATGGTATGTACTCGAACG
For RT-qPCR of the candidate factors	
EF-1α-F	TTCCGAGTTCTATAATGGGTAA
EF-1α-R	ACGCCACAGCTTATTTCTTC
Foxl2-F	GTATCCGCCACAACCTCA
Foxl2-R	CAGGGTCCAAAGTCCAGA
Vtg-F	GGAACTACAACAAATGCGTGAA
Vtg-R	GGCTGTGTATAGATATGCTTG

HSDL2-F	CAGCAAAGACAACAGAGCCACA
HSDL2-R	CTTTCTCCACGGCTGATTGA
Protein singed-F	ACGGTCACAGCAAAGATA
Protein singed-R	TCACTATCCCTGTCCTGTT
CECR-F	CTGTCTGGGACTTATCTTTC
CECR-R	CAGCCTTGTCATAACTCCTA
YBX-F	TTTGTCCATCAGACTGCCATTA
YBX-R	CCTTCCACGACATCAAACTC
TRERF1-F	GGCCTCGTCATGTCGGGTCA
TRERF1-R	TGGTCAGTTAAGGCATACAC
Cf-Uf1-F	GTTGCGGAGACTGGCGAAGA
Cf-Uf1-R	GAAACGCCGTATGAGGGA
Cf-Uf2-F	GAAGGGCAAGCAGTGGTA
Cf-Uf2-R	TAGTCATTTCGTCCTTGC
Cf-Uf3-F	TAGTGCTGCGTTTGCCGTGAT
Cf-Uf3-R	ATCCCGATGTTTGTCCAG
Cf-Uf4-F	CGGGAGGTGATTGACCGAGAA
Cf-Uf4-R	TGATGACTGCGAGGACGA
Cf-Uf5-F	CGTACCAGCCGCTTACACCA
Cf-Uf5-R	AATCATTGGTTTGGCTCCGA

Restriction sites are underlined.

Table S2. Annotation of the positive insert sequences in yeast one-hybrid screening

No	Annotation information	Number of clones
Y1H1	Protein singed	1
Y1H2	Cytochrome P450 1A1	2
Y1H3	Coiled-coil domain-containing protein 92	1
Y1H4	Eukaryotic translation initiation factor 4E transporter	1
Y1H5	Proteasome subunit beta type-4	1
Y1H6	Ependymin-related protein 1	1
Y1H7	Cytochrome c oxidase subunit 6A, mitochondrial	1
Y1H8	Transcription factor BTF3 homolog 4	2
Y1H9	ADP, ATP carrier protein	1
Y1H10	Uncharacterized protein C17orf105 homolog	1
Y1H11	Baculoviral IAP repeat-containing protein 1b	2
Y1H12	Ubiquinone biosynthesis protein COQ4 homolog, mitochondrial	2
Y1H13	10 kDa heat shock protein, mitochondrial	1
Y1H14	Tubulin beta-4B chain	1
Y1H15	Proliferating cell nuclear antigen	1
Y1H16	Probable G-protein coupled receptor 158	1
Y1H17	evm.model.scaffold62981.1 (uncommented factor)	1
Y1H18	evm.model.scaffold64139.1 (uncommented factor)	1
Y1H19	evm.model.scaffold64813.41 (uncommented factor)	1
Y1H20	evm.model.scaffold64999.39 (uncommented factor)	1
Y1H21	evm.model.scaffold36483.59 (uncommented factor)	2
Y1H22	Vitellogenin-4	**4 1**
Y1H23	Guanine nucleotide-binding protein subunit beta-2-like 1	**2 1**
Y1H24	Tubulin alpha-1A chain	**1 1**
Y1H25	Tubulin alpha-1B chain	**2 2**
Y1H26	Ganglioside GM2 activator	**1 3**
Y1H27	Soma ferritin	**1 1**
Y1H28	ATP synthase lipid-binding protein, mitochondrial	**4 1**
Y1H29	Transcription initiation factor TFIID subunit 1	**3 3**
Y1H30	Y-box factor homolog	**2 1**
Y1H31	Transcriptional-regulating factor 1	**2 1**

Y1H32	Nascent polypeptide-associated complex subunit alpha	2
Y1H33	Taurocyamine kinase	1
Y1H34	Hydroxyacyl-coenzyme A dehydrogenase, mitochondrial	1
Y1H35	Heterogeneous nuclear ribonucleoprotein Q	1
Y1H36	Sin3 histone deacetylase corepressor complex component SDS3	1
Y1H37	Histone H2A.v	2
Y1H38	UPF0368 protein Cxorf26 homolog	1
Y1H39	Elongation factor 1-gamma	1
Y1H40	Elongation factor 1-alpha	4
Y1H41	Elongation factor 1-delta	2
Y1H42	Elongation factor 1-beta	1
Y1H43	Arginine/serine-rich protein PNISR	1
Y1H44	Tyrosine-protein kinase BAZ1B	2
Y1H45	Hydroxysteroid dehydrogenase-like protein 2	1
Y1H46	DNA-directed RNA polymerase II subunit RPB4	1
Y1H47	Cat eye syndrome critical region protein 5	1
Y1H48	Serine/arginine-rich splicing factor 4	1
Y1H49	Alpha-ketoglutarate-dependent dioxygenase alkB homolog 4	1
Y1H50	NADH dehydrogenase [ubiquinone] flavoprotein 1, mitochondrial	1
Y1H51	ATP synthase subunit beta, mitochondrial	1
Y1H52	Glyceraldehyde-3-phosphate dehydrogenase	1
Y1H53	Actin, cytoplasmic	2
Y1H54	Superoxide dismutase [Cu-Zn]	1
Y1H55	Mitotic apparatus protein p62	2
Y1H56	Metaxin-3	1
Y1H57	IQ and ubiquitin-like domain-containing protein	1
Y1H58	RNA-binding protein 39	1
Y1H59	Protein translation factor SUI1 homolog	1
Y1H60	Ornithine decarboxylase antizyme 1	1
Y1H61	Zinc transporter ZIP11	1
Y1H62	evm.model.scaffold27357.23 (uncommented factor)	2
Y1H63	evm.model.scaffold61945.9 (uncommented factor)	1
Y1H64	evm.model.scaffold10699.59 (uncommented factor)	1

Blue filling, the first screening; Green filling, the second screening; Orange filling, the cotenant in two screenings: blue font, number of the positive clones in the first screening, green font, number of the positive clones in the second screening.

Table S3. Function and sex difference information of the candidate transcription factors

No	Candidate transcription factors	Function	Number of clones	LogFC	FDR
1	Vitellogenin-4	Lipid transport and storage; Antioxidant activity	4 1	-9.4367	3.49 x 10-38
2	Cytochrome P450 1A1	Participates in the metabolism of various endogenous substrates, including fatty acids, steroid hormones, and vitamins	1	-7.4725	3.26 x 10-3
3	Hydroxysteroid dehydrogenase-like protein 2	Participates in the physiological process of female sex differentiation and the generation and maintenance of secondary sexual characteristics	1	-7.3104	3.26 x 10^{-47}
4	Ganglioside GM2 activator	β -N-acetylgalactosidase activity; Lipid transporter activity	1 3	-5.8531	4 x 10^{-9}
5	Mitotic apparatus protein p62	Participate in the mitotic process	2	-5.1028	9.74 x 10^{-33}
6	Ependymin-related protein 1	Calcium binding	1	-5.0724	9.74 x 10^{-19}
7	Tubulin beta-4B chain	Tubulin is the main component of microtubules	1	-4.5987	1.96 x 10^{-40}
8	Protein singed	Acts as an actin binding protein; It is involved in setae and hair generation, cell differentiation and oogenesis	1	-3.2786	2.08 x 10^{-14}
9	Cat eye syndrome critical region protein 5	Participates in ocular development through the formation of ISWI chromatin complexes	1	-3.0421	5.0 x 10^{-99}
10	Actin, cytoplasmic	Cytoplasm: movement and contraction of cells; Nucleus: regulates gene transcription and movement and repair of damaged DNA	2	-2.8039	2.51 x 10^{-13}
11	Tubulin alpha-1A chain	Tubulin is the main component of microtubules	1 1	-2.071	0
12	Probable G-protein coupled receptor 158	A class of steroid or renal thyroid hormone receptors	1	-1.748	4.0 x 10^{-70}
13	Tubulin alpha-1B chain	Tubulin is the main component of microtubules	2 2	-1.3422	0
14	UPF0368 protein Cxorf26 homolog	------	1	-1.6638	8.0 x 10^{-50}
15	Heterogeneous nuclear ribonucleoprotein Q	Isomeric ribonucleic protein Q	1	-1.3273	3.04 x 10^{-14}
16	Cf-Uf5	------	1	8.2517	5.0 x 10^{-22}
17	IQ and ubiquitin-like domain-containing protein	Participates in cilia formation and maintenance	1	3.9183	0
18	Taurocyamine kinase	Catalyzes phosphate transfer between ATP and various phosphogens	1	3.3264	0
19	Coiled-coil domain-containing protein 92	Participates in adipocyte differentiation	1	3.0652	3.0 x 10^{-22}
20	Histone H2A.v	Mutant histone H2A	2	2.8607	1.0 x 10^{-71}
21	Cytochrome c oxidase subunit 6A, mitochondrial	Cytochrome C oxidase; Participating oxidative phosphorylation	1	1.5183	7.0 x 10^{-12}

22	Arginine/serine-rich protein PNISR	RNA binding	1	1.1457	1.0×10^{-25}
23	RNA-binding protein 39	------	1	1.2183	0
24	Transcriptional-regulating factor 1	Activation of *CYP11A1* transcription; It binds to the progesterone receptor	2 1	1.1161	8.0×10^{-49}
25	ATP synthase lipid-binding protein, mitochondrial	Mitochondrial membrane ATP synthase	1	1.0899	3.0×10^{-49}
26	10 kDa heat shock protein, mitochondrial	Chaperones are involved in the introduction of mitochondrial proteins and the assembly of macromolecules	1	-0.9827	8.0×10^{-48}
27	Elongation factor 1-gamma	Translation extension factor activity	4	-0.6051	4.0×10^{-47}
28	Eukaryotic translation initiation factor 4E transporter	EIF4E binding protein regulates mRNA translation and stability in the processing body (p-body)	1	-0.5465	1.0×10^{-42}
29	Soma ferritin	It has iron oxidase activity	1 1	-0.3918	4.33×10^{-5}
30	Y-box factor homolog	Male gonadal development; Spermatogenesis	2 1	-0.3798	0
31	Superoxide dismutase [Cu-Zn]	Destroy free radicals that are produced in cells and are toxic to biological systems	1	-0.3711	1.33×10^{-9}
32	Tyrosine-protein kinase BAZ1B	Atypical tyrosine protein kinases	1	-0.3434	0
33	DNA-directed RNA polymerase II subunit RPB4	A component of RNA polymerase II that catalyzes the transcription of DNA into RNA	1	-0.3092	2.0×10^{-68}
34	Protein translation factor SUI1 homolog	Participate in the translation process	1	0.9665	3.0×10^{-44}
35	Elongation factor 1-beta	Translation extension factor	1	0.9526	4.0×10^{-89}
36	Arginine/serine-rich protein	------	1	0.9389	1.0×10^{-25}
37	Metaxin-3	Participates in protein transport to mitochondria	1	0.9206	2.0×10^{-50}
38	Ornithine decarboxylase antizyme 1	Ornithine decarboxylase inhibitor activity	1	0.9099	1.0×10^{-33}
39	Glyceraldehyde-3-phosphate dehydrogenase	It has the activity of glyceraldehyde 3-phosphate dehydrogenase and nitrosase	1	0.9062	0
40	Proliferating cell nuclear antigen	Coprotein of DNA polymerase δ	1	0.8529	1.0×10^{-146}
41	Transcription initiation factor TFIID subunit 1	Major components of the TFIID basic transcription factor complex	3 3	0.6598	0
42	Transcription factor BTF3 homolog 4	------	2	0.6563	1.0×10^{-71}
43	ADP, ATP carrier protein	Catalyze the exchange of cytoplasmic ADP and mitochondrial ATP across mitochondrial intima	1	0.6147	0
44	Nascent polypeptide-associated complex subunit alpha	Participates in the synthesis of ribosome-new polypeptide complex	2	0.5489	6.0×10^{-34}

45	NADH dehydrogenase [ubiquinone] flavoprotein 1, mitochondrial	Core subunit of NADH dehydrogenase in mitochondrial membrane respiratory chain	3	0.5461	1.0×10^{-29}
46	Zinc transporter ZIP11	'unctions as a zinc transporter in cells and has zinc ion transmembrane transporter activity	1	0.5278	1.42×10^{-6}
47	Ubiquinone biosynthesis protein COQ4 homolog, mitochondrial	Coenzyme Q is a component of the biosynthetic pathway	1	0.4178	2.0×10^{-82}
48	Guanine nucleotide-binding protein subunit beta-2-like 1	Components of the 40S ribosomal subunit are involved in translation inhibition	2 1	0.2658	0
49	Alpha-ketoglutarate-dependent dioxygenase alkB homolog 4	Protein demethylase	1	0.1772	7.0×10^{-76}
50	Serine/arginine-rich splicing factor 4	Splicing factors in their dephosphorylated form act as general repressors for pre-splicing mRNA	1	0.1142	3.0×10^{-68}
51	Proteasome subunit beta type-4	Endopeptidase activity	1	0	1.0×10^{-111}
52	Baculoviral IAP repeat-containing protein 1b	Cysteine type endopeptidase inhibitor activity is involved in apoptosis	1	0	8.0×10^{-23}
53	Hydroxyacyl-coenzyme A dehydrogenase, mitochondrial	Insulin secretion is controlled by inhibiting the activation of glutamate dehydrogenase 1, which plays an important role in regulating amino acid-induced insulin secretion	1	0	1.0×10^{-147}
54	Sin3 histone deacetylase corepressor complex component SDS3	Regulatory proteins that inhibit histone deacetylase 1 (HDAC1) transcription and enhance histone deacetylase activity	1	0	1.0×10^{-111}
55	Elongation factor 1-alpha	Translation extension factor	4	0	1.29×10^{-3}
56	Elongation factor 1-delta	Translation extension factor	2	0	1.0×10^{-47}

Blue font, number of clones in the first screening; Green font, number of clones in the second screening.

"------", detailed functions are not available.

Log FC=$\text{Log}_2(^{\text{testis expression TPM}}/_{\text{ovarian expression TPM}})$; negative value, highly expressed in ovary; positive value, highly expressed in testis. The greater the absolute value, the greater the expression difference between testis and ovary.

FDR：False discovery rate

Table S4. Relative bioluminescence intensity of *Cf-Foxl2* promoter transcriptional activity detected with DLR

No	Firefly luciferase RLU (F)	Renilla luciferase RLU (R)	Ratio=F/R	Mean
pGL3-Control	10721320	9802	1093.789023	1078.387656
	9908195	9287	1066.888662	
	11573281	10771	1074.485285	
pGL3-Basic	11132	26058	0.42720086	0.575682737
	17969	23634	0.760302953	
	15561	28841	0.539544399	
P0 (-1705/+200)	48820	49247	0.991329421	1.02375612
	50518	47811	1.05661877	
	50288	49142	1.023320174	
P1 (-1705/+1)	62444	45948	1.359014538	1.484931233
	65338	42211	1.54789036	
	59021	38130	1.547888801	
P2 (-1000/+1)/ pGL3-*Foxl2*-Luc	88027	55747	1.579044612	1.548151089
	83536	54681	1.527697006	
	89910	58470	1.537711647	
P3 (-616/+1)	20464	58785	0.348116016	0.357983937
	18967	55264	0.343207151	
	19348	50566	0.382628644	

Table S5. Relative bioluminescence intensity of factors-*Cf-Foxl2* interaction verification detected with DLR

Candidate transcription factors	Firefly luciferase RLU (F)	Renilla luciferase RLU (R)	Ratio=F/R	Mean
pGL3-Control	2648290	3180	832.7955975	878.4109929
	2208204	2530	872.8079051	
	2466307	2653	929.6294761	
pGL3-Basic	15540	27193	0.571470599	0.537531205
	20808	36754	0.566142461	
	10992	23142	0.474980555	
pGL3-*Foxl2*-Luc + pcDNA3.1(+)	18040	26091	0.691426162	0.531996217
	13808	33754	0.409077443	
	11962	24142	0.495485047	
pGL3-*Foxl2*-Luc + pcDNA3.1-*Vtg*	146192	187700	0.778859883	0.796678199
	183789	234755	0.782897063	
	174562	210753	0.828277652	
pGL3-*Foxl2*-Luc + pcDNA3.1-*HSDL2*	286444	192996	1.484196564	1.441310839
	278384	193089	1.441739302	
	267269	191180	1.397996652	
pGL3-*Foxl2*-Luc + pcDNA3.1-*Ps*	309701	240365	1.288461298	1.178757385
	354395	314568	1.126608555	
	346012	308608	1.121202302	
pGL3-*Foxl2*-Luc + pcDNA3.1-*CECR*	236407	142498	1.659019776	1.364194796
	214484	170363	1.258982291	
	173440	147661	1.17458232	
pGL3-*Foxl2*-Luc + pcDNA3.1-*TRERF1*	290484	428726	0.67755163	0.656071027
	345363	593903	0.581514153	
	349616	493009	0.709147298	
pGL3-*Foxl2*-Luc + pcDNA3.1-*YBX*	201350	113374	1.775980384	1.865366463
	194864	104200	1.870095969	
	215850	110691	1.950023037	

	332354	171318	1.939982956	
pGL3-*Foxl2*-Luc + pcDNA3.1-*Uf1*	319601	212752	1.502223246	1.595997717
	285541	212174	1.345786948	
	282939	355519	0.795847761	
pGL3-*Foxl2*-Luc + pcDNA3.1-*Uf2*	258214	305896	0.844123493	0.837145642
	243613	279544	0.871465673	
	307425	208871	1.471841472	
pGL3-*Foxl2*-Luc + pcDNA3.1-*Uf3*	356490	240575	1.481824795	1.378774313
	429548	363206	1.182656674	
	238737	218997	1.090138221	
pGL3-*Foxl2*-Luc + pcDNA3.1-*Uf4*	198985	232411	0.856177203	0.979669915
	274749	276771	0.992694321	
	309701	240365	1.288461298	
pGL3-*Foxl2*-Luc + pcDNA3.1-*Uf5*	354395	314568	1.126608555	1.178757385
	346012	308608	1.121202302	

Figure Legends

Figure S1. Schematic diagram of upstream elements of *Foxl2* core promoter region of Zhikong scallop *C. farreri*

Green line, GC box; Red line, TATA box; Blue line, the octamer frame; ATG, initiation codon at +330; +1, the transcription start site (TSS).

Figure S2. The synthesis, purification and double-strand of cDNA

A. Capillary gel electrophoresis of *C. farreri* total RNA sample; L lane, RNA ladder; 1 lane, *C. farreri* total RNA sample; arrow, 28S rRNA band. B. the purification of mRNA from total RNA. C. the double-strand cDNA synthesized by LD-PCR.

Figure S3. Electropherogram of *C. farreri* total RNA sample.

The visual assessment showed the presence of prominent 28S rRNA peak on RNA sample.

Figure S4. The *C. farreri* gonadal Y1H library construction and quality test

A. Size distribution of full-length cDNA transgenes in the bacteria (primary) library. B. Size distribution of full-length cDNA transgenes in the yeast (secondary) library. C. Plate counting of 100-fold diluted bacteria from the *E. coli* library. D. Plate counting of 100-fold diluted yeast cells from the *S. cerevisiae* library.

Figure S5. The detection of CTNNB1 regulating *Cf-Foxl2* transcription by transient transfection

1: pGL3-*Foxl2-Luc* + pcDNA3.1(+); 2: pGL3-*Foxl2-Luc* + pcDNA3.1-*CTNNB1*; 3: pGL3-*Foxl2-Luc* + pcDNA3.1-*Y-box*. *** $P < 0.001$.

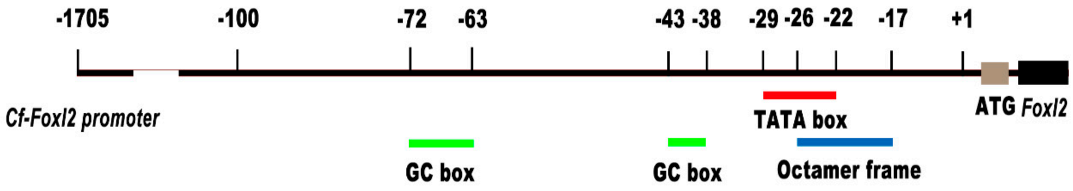
-1705
-100
-72
-63
-43
-38
-29
-26
-22
-17
+1
Cf-Foxl2 promoter
TATA box
ATG Foxl2
GC box
GC box
Octamer frame

Figure S1.

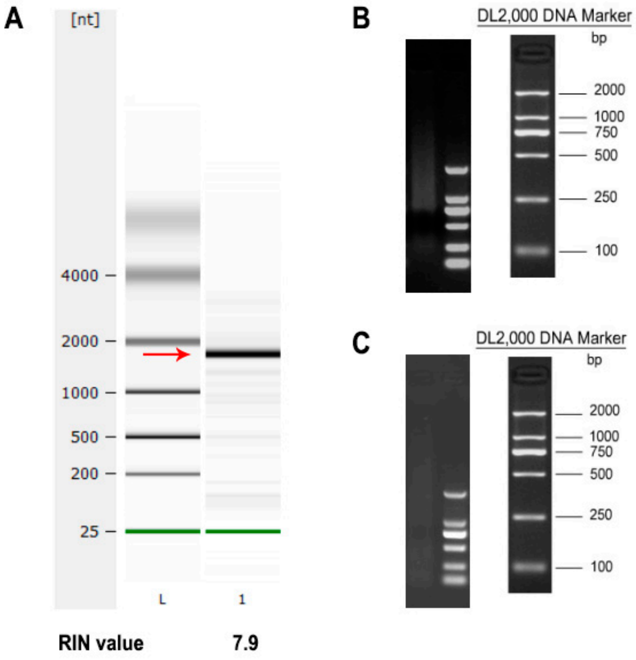
A
[nt]
4000
2000
1000
500
200
25
L
1
RIN value
7.9
B
DL2,000 DNA Marker
bp
2000
1000
750
500
250
100
C
DL2,000 DNA Marker
bp
2000
1000
750
500
250
100

Figure S2.

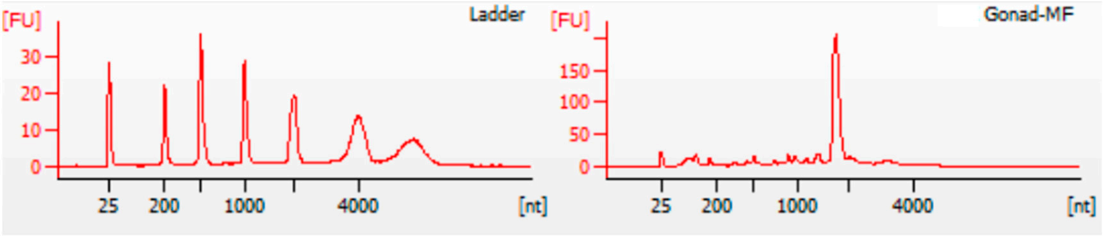

[FU]
Ladder
30
20
10
0
25
200
1000
4000
[nt]
[FU]
Gonad-MF
150
100
50
0
25
200
1000
4000
[nt]

Figure S3.

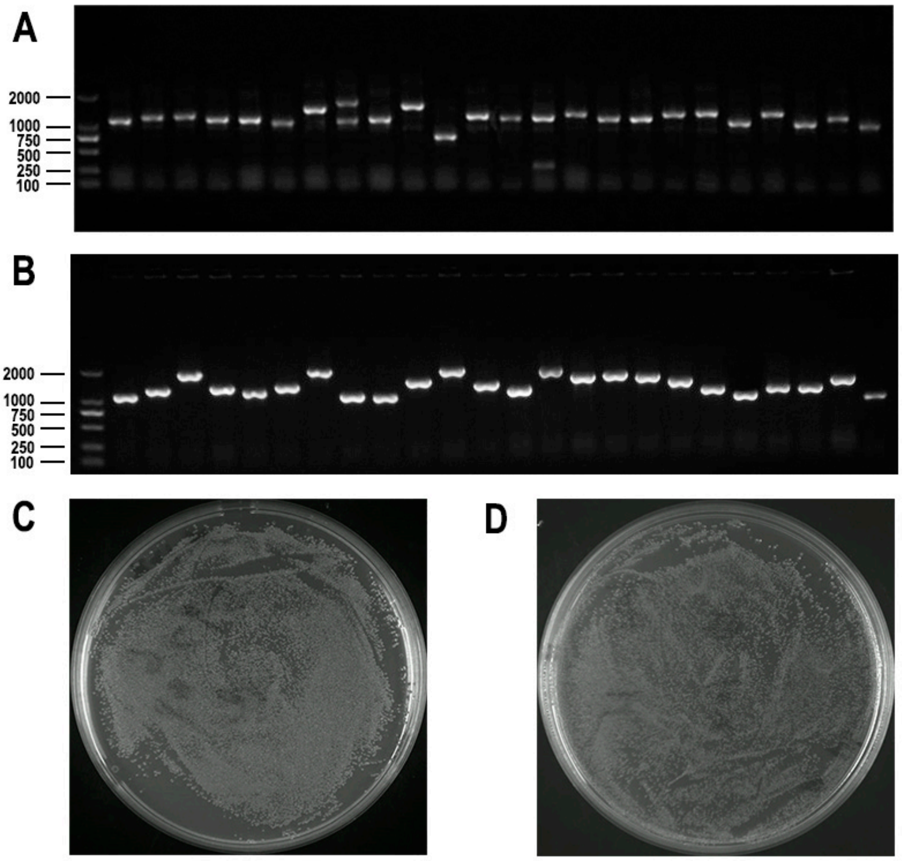

Figure S4.

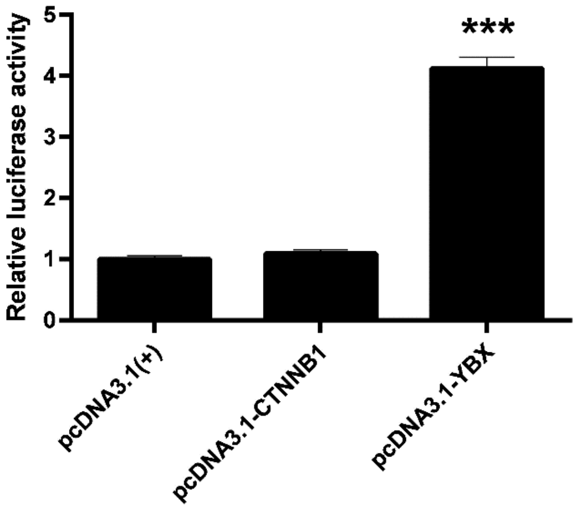

Relative luciferase activity
5
4
3
2
1
0
***
pcDNA3.1(+)
pcDNA3.1-CTNNB1
pcDNA3.1-YBX

Figure S5.
